# Clarifying Nurse–Physician Dual Leadership in Acute Hospital Settings: A Walker and Avant Concept Analysis

**DOI:** 10.1155/jonm/9929355

**Published:** 2026-07-06

**Authors:** Ines Vercalsteren, Filip Haegdorens, Erik Franck

**Affiliations:** ^1^ Workforce Management, Health Systems, and Outcome Research in Care (WORC) Group, Centre for Research and Innovation in Care, University of Antwerp, Antwerp, Belgium, uantwerpen.be

**Keywords:** acute care settings, concept analysis, healthcare professionals, interprofessional collaboration, nurse leaders, nurse–physician dual leadership, physician leaders

## Abstract

**Background:**

Nurse–physician dual leadership (NPDL) has gained attention in healthcare as a model particularly suited to innovation‐driven environments, where the complex demands of leadership may outpace the capacities of a single leader. By combining the distinct yet complementary expertise of nurses and physicians, NPDL offers a hybrid structure that balances the clarity of single leadership with the breadth of shared leadership. Despite its growing presence in healthcare, the concept remains insufficiently defined in the literature.

**Aim:**

To clarify the concept of NPDL in acute hospital settings, ensuring consistent terminology and providing a solid foundation for further research, education and practice.

**Methods:**

Concept analysis guided by the Walker and Avant 8‐step method. A systematic literature search was conducted through PubMed, ScienceDirect and Embase. Keywords included co‐leadership, dual leadership and dyad leadership, with and without the prefix ‘nurse–physician’ and combined with healthcare‐related terms. Peer‐reviewed articles, books and grey literature, including definitions, attributes, antecedents, consequences and empirical referents, were identified and managed using EndNote. Of 247 identified articles, 23 were included, with eight additional sources retrieved through manual reference list searching.

**Results:**

A definition of NPDL in acute hospital settings is proposed. Four defining attributes were identified: formalised dual leadership structure, joint responsibility, joint decision‐making and role complementarity. The analysis also identified five antecedents that were categorised into organisational and relational factors. The consequences of NPDL include stronger leadership, enhanced interprofessional collaboration, improved decision‐making and the presentation of a united leadership front.

**Conclusion:**

NPDL can be defined as a collaborative leadership model in which two formal leaders, a nurse and a physician leader, work together as co‐leaders, with different skill sets and without a power difference, and they are jointly accountable and responsible for the organisational or unit results.

**Impact:**

Clarifying the concept of NPDL enhances future research. It could inform policymakers and support both educators, who provide formal leadership training, and facilitators, who guide professional development through coaching and mentoring, in fostering effective collaboration between nurse and physician leaders.

**Patient or public involvement:**

This study did not include patient or public involvement in its design, conduct, or reporting.

**Contribution to the field**
• This provides conceptual clarity on NPDL, enabling consistent use and understanding across clinical and academic settings.
• Identifies the defining attributes, antecedents and consequences of NPDL, offering a foundation for future empirical research and leadership development.
• Supports clinical leaders, educators and policymakers in implementing effective co‐leadership models that strengthen interdisciplinary collaboration and improve patient care outcomes.

## 1. Introduction

Effective leadership is crucial for delivering high‐quality, cost‐effective care in hospitals. In today’s complex and rapidly evolving healthcare environment, leaders are increasingly expected to spearhead initiatives that enhance care outcomes while efficiently managing resources. The shift from traditional reimbursement models towards value‐based care has further emphasised this need [1, 2]. As leadership demands exceed the capacities of any single individual, the notion of Drucker [3]—that reliance on a solitary leader is problematic—gains renewed relevance, underscoring the potential of dual leadership structures in today’s healthcare landscape.

Dual leadership, in which two individuals share leadership responsibilities, is prevalent in various industries (e.g., banking, schools and arts) [4–6]. It is considered particularly well‐suited to innovation‐driven environments, such as healthcare, where complex demands may outpace the capacity of a single leader [7]. In healthcare, dual leadership—particularly between nurses and physicians—is increasingly adopted to leverage the distinct yet complementary expertise of two key stakeholders in the healthcare system [8–12]. This approach addresses the limitations of siloed (clinical) leadership by fostering collaboration across disciplines, thereby supporting cohesive clinical, operational and strategic decision‐making [1, 2]. It offers a hybrid structure that balances the clarity of single leadership with the breadth of shared leadership [13]. Yet, despite its growing presence in healthcare, the concept remains insufficiently defined in the literature.

Nurse–physician dual leadership (NPDL) may be implemented at various organisational levels [9, 12, 14, 15]. It applies to both the organisation and management of acute hospital services as well as the coordination of clinical care. Although the specific responsibilities differ across organisational levels, the model encompasses both strategic and operational leadership roles shared between nurse and physician leaders. At the strategic level, collaboration between Chief Nursing Officers (CNOs) and Chief Medical Officers (CMOs) shapes the institutional vision and policy [12]. At the departmental and operational levels, dual leadership supports integrated care and shared decision‐making in day‐to‐day clinical practice [4, 14].

Several countries have adopted dual leadership models, in which a nurse and a physician leader share responsibilities, for example, Denmark [4], the United States [16, 17] and Canada [18, 19]. Although nurses and physicians differ in values and styles [12], the executive nurse leader is considered the ideal complement to partner and collaborate with the physician leader [16]. Additionally, recent literature also demonstrates growing interest in this leadership model as a promising approach to strengthen interdisciplinary collaboration, with professional bodies such as the American College of Cardiology actively advocating for its adoption [20]. This trend is further supported by a recent narrative review that re‐evaluates the leadership model and highlights its contemporary relevance [21].

According to Denis et al. [22], dual leadership aligns with the second stream of ‘leadership in the plural’, where leadership capacities are shared among those at the top of the organisation. Such co‐leadership structures are particularly relevant in knowledge‐intensive sectors, such as healthcare, where a team of leaders jointly lead others, offering resilience and multidimensional perspectives [22, 23]. This plural leadership perspective provides a theoretical rationale for NPDL in clinical practice. Acute hospital settings can be understood as complex adaptive systems (CASs), in which outcomes emerge from the interactions of multiple professional groups [24, 25]. Such systems are characterised by unpredictability, ambiguity and nonlinearity [24]. Acute hospital settings are associated with high uncertainty, rapid decision‐making, and the need to integrate diverse forms of professional knowledge [25]. Within such contexts, leadership models that combine different perspectives and complementary expertise and skills are considered particularly appropriate [14]. Shared leadership at the top enables the combination of nursing and medical expertise in strategic and operational decision‐making [14, 26]. It may enhance coordination of care, patient safety, quality outcomes and team and organisational outcomes [14, 26]. From this perspective, NPDL can be understood as a concrete manifestation of ‘leadership in the plural’ within the acute hospital setting as a CAS.

These contextual features reflect the realities of acute hospital care, where professional differentiation, high interdependence, and time‐critical decision‐making shape leadership demands [24]. Although organisational structures vary across health systems and between public and private hospitals [16, 18], the fundamental clinical dynamics of acute hospital care remain largely comparable and require effective interprofessional collaboration to ensure safe and high‐quality patient care [27]. Consequently, leadership approaches that facilitate effective nurse–physician collaboration, such as NPDL, are likely to be relevant across different organisational contexts.

Within these acute hospital settings, nurses and physicians operate within distinct professional and legal scopes of practice, with nurses ensuring continuous care coordination and operational flow, while physicians hold authority over diagnosis and treatment [28]. These parallel structures create dual lines of responsibility that rarely converge at the leadership level, reinforcing traditional hierarchies and siloed decision‐making. Although these structures are increasingly being challenged by interprofessional and collaborative care models, differences in attitudes towards interprofessional collaboration between physicians and nurses persist [29]. Introducing NPDL directly addresses this structural separation by establishing shared responsibility and complementary leadership roles and skills [14], aligned with the interdependent and time‐critical nature of acute care across both public and private hospital systems [16].

Despite the growing interest in NPDL in healthcare, the literature remains inconsistent in its terminology and interpretation. Terms such as *co-leadership*, *dyad leadership*, *dual leadership,* and *productive pairs* are used interchangeably—within and outside the healthcare context—to describe similar leadership structures. Moreover, the same term is often interpreted differently by various authors, leading to misconceptions and assumptions that may impede research and cause confusion among educators and professionals.

**FIGURE 1:**
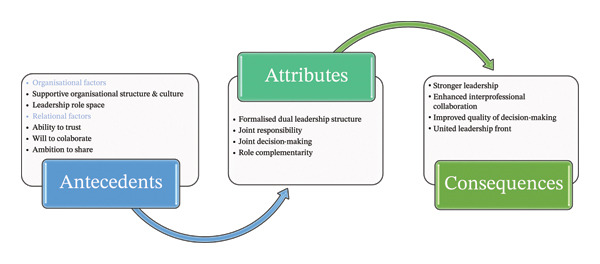
Defining attributes, antecedents and consequences of nurse–physician dual leadership in acute hospital settings.

Based on a comprehensive search of accessible healthcare databases, concept analyses in nursing leadership have been published [30], although no concept analysis specifically addressing NPDL in acute hospital settings has been identified. NPDL remains insufficiently defined and inconsistently used across the literature, and no universally accepted definition exists within the health sciences. Clarifying this concept is essential for researchers to advance theory and build comparable evidence, and for policymakers and practitioners it is essential to fully leverage the potential of NPDL. This concept analysis aims to provide a clear definition and a conceptual model of NPDL, using the Walker and Avant method. Figure 1 illustrates the resulting conceptual model, including the key attributes, antecedents and consequences of the concept.

## 2. Background

Reinforcing the perspective introduced by Peter Drucker in 1954—who argued in The Practice of Management that ‘90% of the trouble we have with the chief executive’s job is rooted in our superstition of the one‐man chief’ [3]—Gibb [31] was among the first scholars to conceptualise leadership as a collective rather than an individual phenomenon. He proposed that leadership is best understood as a group attribute, enacted and distributed across members of a group.

A decade later, Etzioni [32], drawing on small group research, argued that task‐orientated teams require both expressive (social‐emotional) and instrumental (task‐focused) leadership, ideally enacted by two mutually supportive individuals. Hodgson et al. [33] similarly found that two leaders can divide tasks between external and internal organisational roles.

Among other theoretical insights, these inspired practice. In 1988, a Toronto academic hospital implemented a co‐leadership model at the unit level, based on three principles: increased responsibility closer to the patient, clear accountability for decision‐making, and direct involvement of physicians in management [34]. As such models emerged in practice, scholars continued refining theoretical frameworks—often using varying terminology. Gilmore [35] described ‘productive pairs’, a model based on partnerships between individuals from complementary disciplines who share common goals, Pearce [36] advocated for shared leadership in complex environments, and Steinert et al. [26] positioned it as central to effective hospital management, aligning with the aims of the European Foundation for Quality Management.

Denis et al. [22] reviewed studies on plural forms of leadership, highlighting a shift away from traditional ‘heroic’ models of unitary leadership towards more distributed and collective approaches. Still, research on dual leadership remains limited [4, 37], and there is a notable absence of studies examining the impact of a collaborative relationship between nurse and physician leaders at the patient care unit level.

Steinert et al. [26] argued that a systematic evaluation of the effect of changes in hospitals and health services’ management and leadership structures on quality of care and cost‐effectiveness should be an objective for further research. Despite its increasing adoption, there is a paucity of empirical studies in peer‐reviewed literature, and this topic is more widely discussed in the grey literature [19].

### 2.1. Aims

The purpose of this concept analysis is to clarify the concept of NPDL to ensure its consistent and accurate use in research, education and practice. By identifying and distinguishing between the various synonyms and interpretations found in scientific discourse, this study seeks to establish a clear and shared understanding of NPDL. This conceptual clarity is essential for advancing research in this topic and could be of importance when implementing NPDL in clinical practice.

## 3. Methods

This concept analysis was guided by the Walker and Avant method, following their ‘*Theory Construction in Nursing framework’* [38]. This approach was chosen for its widespread acceptance in nursing research and its systematic and transparent methodology.

**FIGURE 2:**
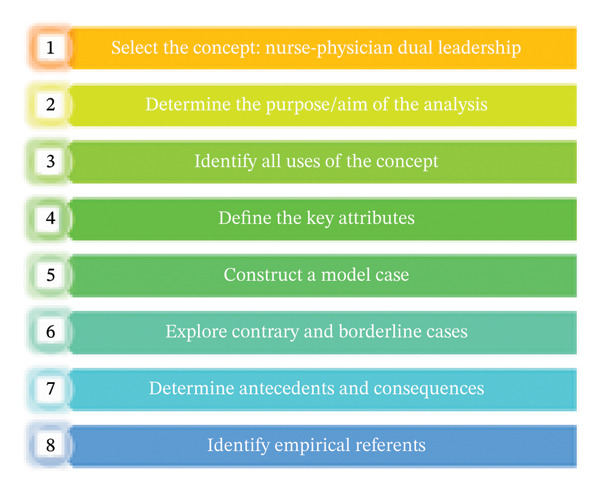
Walker and Avant’s 8‐step method of concept analysis.

As shown in Figure 2, the analysis adhered to Walker and Avant’s 8‐step process, which includes (1) selecting the concept; (2) determining the purpose or aim of the analysis; (3) identifying all uses of the concept; (4) defining its key attributes; (5) constructing a model case; (6) exploring contrary and borderline cases; (7) determining antecedents and consequences; and (8) identifying empirical referents [38].

### 3.1. Data Sources

#### 3.1.1. Search Strategy

An initial literature search was performed using three databases: PubMed, ScienceDirect and Embase. To clarify the concept and to develop a working definition, as well as to describe the antecedents, attributes and consequences, a structured literature search was conducted. Articles were searched using the following search terms in the title or abstract fields: *nurse–physician leadership*, *nurse–physician co-leadership, nurse–physician dual leadership, nurse–physician dyad leadership, co-leadership, dual leadership, dyad leadership,* combined with contextual terms such as healthcare, health‐care, hospital, hospitals, acute hospital, acute hospitals and hospital setting*.* These terms were selected based on their conceptual proximity and their usage in the existing literature, as they are potentially used to describe the same underlying phenomenon. Other related search terms, such as ‘shared leadership’, ‘collective leadership’, ‘collaborative leadership’ and ‘interprofessional leadership’, were initially considered. However, preliminary searches using these terms generated an overwhelming number of results that were too broad or only tangentially related to the specific concept of NPDL. To maintain a clear focus and ensure the relevance of the literature, these broader terms were excluded from the final search strategy.

No restrictions were placed on publication year, as both recent and older articles may provide valuable insights and offer historical context to the concept analysis. Similarly, no limitations were applied regarding the geographical origin of the publications, ensuring a comprehensive, global perspective on the topic. Inclusion criteria were articles published in or translated into English and studies focused on healthcare. Broader leadership terms (co‐leadership, dual leadership and dyad leadership) also identified some context‐independent literature. These articles were included only if they provided a relevant theoretical contribution to NPDL, ensuring consistency with our focus on acute hospital settings. Exclusion criteria were studies outside the healthcare sector, inter‐organisational leadership models and literature not addressing formal nurse–physician leadership. The analysis focused exclusively on formal leadership roles with hierarchical responsibilities within a single organisational context. Screening was further conducted in two stages: first by title, then by abstract and performed exclusively by the primary author. All references were managed using EndNote, which facilitated organisation and duplicate removal throughout the selection process. In cases of uncertainty, inclusion decisions were discussed with the research team until consensus was reached. An additional search involved backward citation screening, where the reference lists of selected articles were examined to identify additional relevant sources. The literature included peer‐reviewed journal articles, scholarly books, and grey literature such as editorials if they were considered relevant to the objectives of this concept analysis. The grey literature, including editorials and reports, was included to capture emerging perspectives and practice‐based insights, not always present in peer‐reviewed journals, in line with recommended practices for integrative reviews. It also provides additional contextual information that can further clarify the concept [39]. The quality of these sources was appraised during screening, and only those considered credible and relevant to NPDL were included. The retrieved articles were then screened for relevance based on their potential contribution to defining the concept of NPDL. For the body of the concept analysis, the focus was primarily on literature within the healthcare sector regarding the definition, attributes, antecedents and consequences.

Several limitations are inherent to the search strategy. First, only three databases (PubMed, ScienceDirect and Embase) were searched, which may have resulted in relevant studies being omitted. Second, only publications in English were included, introducing potential language bias. Third, broader but related leadership terms (e.g., shared, collective, collaborative and interprofessional leadership) were initially considered but excluded to maintain conceptual specificity, which may have led to some studies being missed. Nonetheless, studies employing alternative terminology were captured through snowballing. Finally, grey literature was included, which may introduce variability in quality but provides valuable contextual and practice‐based insights relevant to concept clarification. To ensure transparent and comprehensive reporting of the literature search and selection process, this review was conducted and reported in accordance with the Preferred Reporting Items for Systematic Reviews and Meta‐Analyses (PRISMA) statement [40].

#### 3.1.2. Search Outcome

**FIGURE 3:**
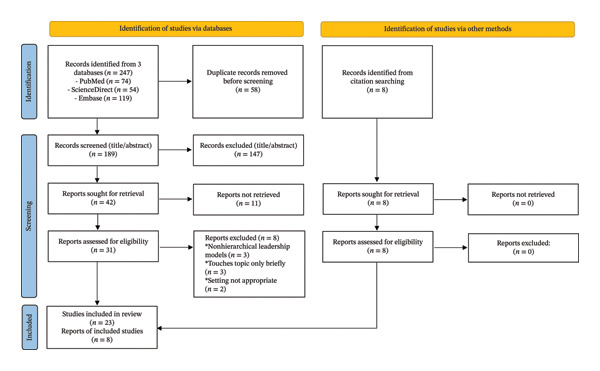
PRISMA flow diagram of NPDL (data search and selection process).

The initial search yielded 247 citations, encompassing research across all sectors. After screening by title and abstract, all duplicates were removed. Articles that were not relevant to the concept of NPDL were excluded. In addition, research focusing on industry, banking, the arts, education and aviation was removed to create a focus on health‐related contexts. This resulted in 42 articles that were assessed based on their full texts. Subsequently, 23 articles were retained based on their full‐text assessment. Manual searching from the reference lists of relevant articles (snowballing) was also conducted, leading to the inclusion of one book and seven additional articles. Data sufficiency was considered reached when additional searches and snowballing no longer yielded new attributes, antecedents, or consequences relevant to NPDL. After repeated searches returned only studies that overlapped conceptually with those already included, no further unique contributions were identified, indicating conceptual saturation. Figure 3 shows the overall process, summarised in the PRISMA flow diagram of NPDL, which was adapted from Page et al. [40].

## 4. Results

### 4.1. Identifying All Uses of the Concept

The third step in the Walker and Avant method is to identify all uses of the concept [38]. Several data sources were systematically searched to explore what is currently known and unknown about the concept. In line with this methodology, a broad exploration of the concept was undertaken, including its use across disciplines such as healthcare, management and organisational studies, drawing on dictionaries, international databases and grey literature.

#### 4.1.1. Dictionaries

NPDL is a complex concept and was therefore deconstructed into its constituent components: *nurse*, *physician*, *dual* and *leadership*.

According to the Cambridge University Press online dictionary, ‘nurse’ is defined as ‘a person whose job is to care for people who are ill or injured, especially in a hospital’ [41], while a ‘physician’ is defined as ‘a medical doctor, especially one who has general skills and is not a surgeon’ [42].

While the Cambridge dictionary defines ‘dual’ as ‘with two parts, or combining two things’ [43], the related term ‘duality’, defined as ‘the state of combining two different things’, even better reflects the integration of two distinct yet complementary perspectives, which lie at the core of NPDL. To enhance clarity, the authors provide the following example: ‘duality can be exploited to solve problems by considering simultaneously two perspectives, the primal and dual view of the problem’ [44]. Finally, a ‘leader’ is defined as ‘a person in control of a group, country or situation’ [45], whereas ‘leadership’ refers, on the one hand, to the position or fact of being the leader and, on the other hand, to the set of characteristics that make someone a good leader [46].

#### 4.1.2. International Online Databases

Dual leadership is prevalent across a variety of contexts, including banking, journalism and high‐tech business [4]. It has been studied and adopted in school contexts and is a common structure in arts organisations [5, 6]. Beyond its application across sectors, dual leadership is also conceptualised within broader leadership theories. ‘Leadership in the plural’ is a broad term that refers to different approaches of multiple individuals exhibiting leadership behaviour. Denis et al. identified four streams of scholarship on plural leadership. One of these streams focuses on pooling leadership at the top of organisations. This stream emphasises the pooling of leadership capacities at the top to direct others [22]. This can be two, three, or more people who jointly work together as co‐leaders of others outside the group [23, 33].

Although the included studies use a variety of terms to describe this phenomenon—such as dyadic leadership, co‐leadership, professional duo, or productive pair—this analysis consistently uses the term NPDL. This terminological choice was made to enhance conceptual clarity and ensure readability throughout the text.

Table 1 summarises the studies included in this analysis, outlining the authors’ definitions and interpretations of the concept. This overview demonstrates the variation in terminology, yet all refer to the same underlying phenomenon. By adopting a single, clearly defined term, this concept analysis aims to reduce confusion and provide a coherent framework for discussion.

**TABLE 1:** Details on identified research.

Author	Title	Year	Discipline	Country	Methodology	Main findings	Definition of dual leadership (DL) or synonym
Alvarez, J.L.; Svejenova, S.	Sharing Executive Power. Roles and Relationships at the Top.	2005	Strategic management	United Kingdom	Book Chapter (Chapter 4)	Not applicable	The professional duo consists of two executives who, over time, perform the top job together in a coordinated fashion and are held jointly accountable for the company or unit’s results.
Belasen, A.T.; Belasen, A.M.; Belasen, A.R.; Belasen, A.R.	A win‐win for health care: promoting co‐leadership and increasing women’s representation at the top	2021	Healthcare	United States	Conceptual paper that reviews the evidence that when single‐leadership models are used and women are underrepresented in leadership, the healthcare industry may miss out on opportunities to increase efficiency and quality of care.	This paper describes a co‐leadership model with distinct and overlapping roles, which promotes women’s participation and inspires administrative and clinical leaders to collaborate and achieve optimal performance. The dyad as the enabling track for women in healthcare leadership creates opportunities for healthcare systems to bridge the gender gap in senior positions as well as improve the delivery of cost‐effective quality care.	Co‐leadership is a form of interprofessional cooperation or shared leadership between two or more individuals with complementary strengths and abilities. Each co‐leader takes on distinct, though often overlapping leadership roles.
Biga, C.	Synergizing Success: The Power of Dyad Leadership in Cardiology	2024	Nursing, medicine	United States	Expert opinion	This article explores the top benefits, challenges and opportunities for the future of dyad leadership in cardiovascular care. The author concludes that dyad leadership offers a compelling opportunity to improve interdisciplinary collaboration, foster innovation and potentially redefine the landscape of cardiovascular care. Successful implementation will require stakeholders to encourage and support this collaborative model. It is key to invest in training programs that equip healthcare professionals with the skills and competencies needed to thrive within dyad structures.	Dyad leadership is described as a leadership structure that typically allows for a physician and a nonphysician administrator to share responsibility for strategic and operational oversight.
Chazal, R. A.; Montgomery, M. J.	The Dyad Model and Value‐Based Care	2017	Healthcare	United States	Expert opinion	This article discusses the implementation of the dyad leadership model, pairing a clinical leader with an administrative leader, as a strategy to enhance collaboration, align clinical and operational goals and improve patient care within healthcare systems. The article provides insights based on the authors’ experiences, particularly within the Lee Health System in Florida, highlighting the benefits and considerations of adopting this leadership approach in the context of value‐based care.	No explicit or formal definition of dyad leadership is provided in this article
Clark, R. C.; Greenawald, M.	Nurse‐physician leadership: insights into interprofessional collaboration	2013	Nursing, medicine	United States	Qualitative research to identify themes characterizing collaboration from the perspectives of nurses and physicians serving in complementary leadership roles in intensive and progressive care hospital units.	The study highlights the importance of organisational support, shared expectations, strong relationships and effective communication in fostering collaboration between nurses and physicians. It underscores the need for deliberate and structured interprofessional communication to strengthen teamwork and advance collaborative practice.	No explicit or formal definition of nurse–physician leadership is provided in this article
Clausen, C.; Lavoie‐Tremblay, M.; Purden, M.; Lamothe, L.; Ezer, H.; McVey, L.	Intentional partnering: a grounded theory study on developing effective partnerships among nurse and physician managers as they co‐lead in an evolving healthcare system	2017	Nursing, Medicine	Canada	Grounded theory	The grounded theory of ‘intentional partnering’ explains how nurse and physician managers align their professional agendas to benefit from their partnership. Key processes include accepting mutual necessity, daring to risk together and constructing shared responsibility, which enable effective collaboration focused on patients’ interests	A formal nurse–physician management dyad was operationally defined as a nurse and physician who have a formal management position in the organisation; have been appointed to work together to manage and lead a program, department or division of care; are known to each other and periodically come together to discuss clinical management issues
Clouser, J. M.; Vundi, N. L.; Cowley, A. M.; Cook, C.; Williams, M. V.; McIntosh, M.; Li, J.	Evaluating the clinical dyad leadership model: a narrative review	2020	Nursing, medicine	United States (Kentucky)	Narrative review for evaluating dyad leadership models in health systems	Evidence for implementing a dyad leadership model in the healthcare setting is limited. The review highlights several potential organisational advantages to implementing dyadic leadership models.	The authors refer to the definition of Chazal and Montgomery (2017). Clinical dyads (i.e., nurse/physician or clinician/administrator) represent a specific type of shared leadership, in which the leadership is not only shared among two individuals, but they also hold different expertise, training, credentials and perspectives.
Collins, S.; Jacobs, J.; Perryman, R.	Dyad Leadership: 1 + 1 ≥ 2	2016	Healthcare	United States	Conceptual paper	Not applicable	Dyad management is a model of formal leadership in which two individuals with different skill sets, educations, and backgrounds, are paired to better fulfil the mission of the organisation. In the management of a medical institution, the paired leaders are often a physician partnered with an administrator.
Etzioni, A.	Dual leadership in complex organisations	1965	Sociology	United States	Theoretical article	Etzioni introduces the concept of dual leadership in complex organisations, drawing on the Bales–Parsons analysis of small groups. He distinguishes between expressive (socio‐emotional) and instrumental (task‐oriented) leadership, arguing that these complementary roles are best fulfilled by two individuals. A single leader cannot effectively manage both social and technical demands, making a dual leadership structure more suitable for complex organisations structure, where two leaders share responsibility for different aspects of the organisation.	No explicit or formal definition of dual leadership is provided in this article
Fjellvaer, H.	Dual and unitary leadership: Managing ambiguity in pluralistic organisations.	2010	Strategic management	Norway (Bergen)	Qualitative research with in‐depth interviews with 63 leaders in 27 organisations. Respondents came from hospitals, colleges and universities, museums, orchestras, theatres and newspapers.	Dual leadership is quite common in certain types of organisations, but advocates of the chain‐of‐command format still regard it as a recipe for disaster and view unitary leadership as the only alternative. Dual leadership is a viable option for those who see their organisations as consisting of two or more different worlds.	Dual leadership is a situation wherein two persons of roughly equal rank divide the executive leadership roles and functions between them.
Gerardi, D.	Synergy as strategy: a model for clinical partnering	2018	Healthcare	United States (California)	Practice‐based conceptual article	Not applicable	The author uses the definition of Sanford and Moore (2015): dyads are mini‐teams of two people who work together as co‐leaders of a specific system, division, clinical service line or project.
Gilmore, T.N.	Briefing notes: Productive pairs.	1999	Organisational science	United States (Philadelphia)	Briefing notes	Not applicable	No explicit or formal definition of dual leadership is provided in this article
Hunter, S.; Allen, J.B.; Heinen, R.; Cushenbery, L.	Chapter 12: Proposing a Multiple Pathway Approach to Leading Innovation: Single and Dual Leader Approaches	2018	Organisational science	United States	Book chapter (Chapter 12)	Not applicable	Dual leadership is a collective approach where two individuals share the leadership workload and through coordinated efforts, engage subordinates toward the accomplishment of a set of the often‐disparate goals characterising the generation and implementation of novel ideas
Hunter, S.; Cushenbery, L.; Cushenbery, D.; Bradley, J.	Why dual leaders will drive innovation: Resolving the exploration and exploitation dilemma with a conservation of resources solution	2017	Organisational science	United States (Pennsylvania)	Theoretical article	Not applicable	Dual leadership is a collective approach where two individuals share the leadership workload and through coordinated efforts, engage subordinates toward the accomplishment of a set of the often disparate goals characterizing the generation and implementation of novel ideas.
Iannazzo, A.; Lorenz, H.; McLaughlin, M.	The Executive Nurse Leader in Service Line Management:: An Experience of a Hospital Health System	2019	Nursing, medicine	United States (Pennsylvania)	Descriptive case study	The article describes the unique experience of a system hospital and the successes of the executive nurse leader as the co‐lead of the physician leader in service line management. The authors state that dyad leadership in service line management requires leadership skills that only the physician and nurse can create synergistically. Although other clinical professionals might be equality suitable for operational or financial management, no other professional has been trained in teamwork, communication and collaboration like the nurse. The nurse is consistently successful working alongside the physician, originating at the bedside and extending through all domains, including successful service line management as exemplified by the experiences at UPMC.	The dyad is traditionally a physician and nonphysician co‐leader that oversees integrated clinical service lines, divisions of care providers or entire community service delivery systems.
Janssens, S.; Clipperton, S.; Simon, R.; Lowe, B.; Beckmann, M.; Marshall, S.	Clinicians’ attitudes towards a co‐leadership structure for maternity emergency teams: An interview study.	2022	Midwifery, medicine	Australia (Queensland)	Qualitative interview study addresses the gaps in knowledge regarding clinician attitudes toward co‐leadership and how a co‐leadership structure might be implemented within a maternity care setting. Twenty‐five clinicians working in the birthing units of two tertiary maternity units were interviewed and a conventional content analysis conducted.	Clinicians viewed co‐leadership as potentially beneficial to patient care through improved leadership performance and co‐leader back up behaviour. Implementation was thought to require a supportive organisational culture, agreed patient management protocols and the participation in simulation training.	This authors use the definition of (Pearce and Conger, 2002): Co‐leadership is a specific form of planned rather than emergent shared leadership consisting of two individuals sharing leadership tasks in a complimentary and interdependent fashion.
Jennings, B. M.; Disch, J.; Senn, L.	Patient Safety and Quality: An Evidence‐Based Handbook for Nurses. Chapter 20. Leadership	2008	Nursing, medicine	United States (Maryland)	Brief history of the concept of partnered leadership and a description of one specific study	A notable absence of research on the impact of a collaborative relationship between the nurse and physician co‐leaders of patient care units. They describe the concept of partnered leadership from the perspective of three professional healthcare organisations. The authors discuss the concept of productive pairs, which was developed by Gilmore (1999). Eventually they highlight a study from Tjosvold and Mac Pherson about the way in which physician leaders and nurse administrators worked together in managing areas within the hospital.	Productive pairs (Gilmore, 1999) is defined as a leadership model that is based on a partnership between two individuals who share common goals and come from different, yet complementary, disciplines.
MacTavish, M.; Norton, P.	Redesign of a health science centre: reflections on co‐leadership.	1995	Nursing, medicine	Canada (Toronto)	Brief report	Sunnybrook hospital in Toronto initiated a trial of management change in 1988 and a major re‐engineering effort beginning in 1992 that aimed to decentralize management. The initiative focused on implementing a co‐management model with shared leadership responsibilities, particularly involving physicians in decision‐making. This model was guided by 3 principles: shifting responsibility closer to the patient, maintaining a centralized structure and ensuring clear accountability. The hospital implemented a co‐leadership model for the lead management of levels	The vice‐president co‐leadership model involves a team of two for each clinical unit: a vice‐president operations and a vice‐president medical. Both have the same job description and reporting structures. They are jointly responsible for ensuring that re‐engineering takes place and are required to be committed to the major culture change that this implies. Therefore, the chief operating officer, to whom they both report, requires joint goals and objectives for their annual performance appraisal.
Nguyen, T.L.; Hunter, S.	Chapter 6 ‐ Shared leadership arrangements for creativity and innovation	2023	Organisational science	United States	Book chapter	Not applicable	Dual leadership is a form of semivertical leadership that is characterised by both lateral influence between coleaders and downward influence on followers.
O′Leary et al.	Use of Unit‐Based Interventions to Improve the Quality of Care for Hospitalized Medical Patients: A National Survey	2017	Nursing, medicine	United States	Cross‐sectional survey to characterize the use of unit‐based interventions that aim to improve the quality of patient care	The authors found variation in the use of unit‐based interventions intended to improve the quality of care for medical inpatients.	Unit nurse–physician co‐leadership is a collaborative model in which a nurse manager and a physician medical director share responsibility for quality on their unit.
Patton, P.; Pawar, M.	New clinical executive models: one system′s approach to chief nursing officer‐chief medical officer co‐leadership.	2012	Nursing, medicine	United States	Descriptive case study	The authors find that co‐leadership enhances alignment and integration, whereby shared goals and mutual accountability are critical and leadership development is essential for success.	No explicit or formal definition of co‐leadership is provided in this article
Ponte, P. R.	Nurse ‐ physician co‐leadership. A model of interdisciplinary practice governance	2004	Nursing, Medicine	Massachusetts	Editorial	Not applicable	No explicit or formal definition of co‐leadership is provided in this article
Sanford, K.; Moore, S.	Dyad leadership in healthcare: When one plus one is greater than two	2015	Nursing, medicine	United States (Colorado)	Book	Not applicable	Dyads are mini‐teams of two people who work together as co‐leaders of a specific system, division, clinical service line, or project. Dyad management is a model of formal leadership in which two individuals with different skill sets, education, and backgrounds are paired to better fulfil the mission of the organisation. The two partners have different job descriptions, and different duties that complement each other. Their combined skills complete a set of management competencies needed for accomplishing particular clinical, business and strategic goals of the organisation.
Saxena, A.	Challenges and success strategies for dyad leadership model in healthcare	2021	Nursing, medicine	Canada	Mixed‐method: focus‐groups, surveys and semistructured interviews, perceptions of 37 leaders in 1 HCO at different hierarchical levels were analysed on their lived experiences	The challenges and success strategies spanned personal, interpersonal and organisational domains. The areas requiring attention included mindsets, competencies, interpersonal relationships, support, time, communication and collaboration. Also, the importance of organisational context was highlighted.	Dyad leadership in healthcare refers to a Physician (PL) and another leader, Dyad Co‐leader (DCL) with a different background, sharing responsibilities for leading an organisation or its components. ‘Dyads’ are mini‐teams of two people who work together as co‐leaders of a specific system, division, clinical service line or project.
Saxena, A.; Davies, M.; Philippon, D.	Structures of health‐care dyad leadership: an organisation’s experience.	2018	Nursing, medicine	Canada	Mixed‐method: the perception of 32 leaders in formal leadership positions through focus groups and surveys, in addition 5 senior leaders were interviewed	The authors aim to explore the structural aspects (role, responsibilities and reporting) of dyad leadership in one healthcare organisation. There are a large number of shared responsibilities that spans the leadership (e.g., global performance and quality improvement) and management (e.g., human resources, budgets and education delivery) domains. The individual responsibilities, except for staff and physician engagement, are in the management domain. Both partners are responsible for joint decision‐making, projecting a united front and joint reporting through a quadrat format. The mutual relationship and joint accountability are key characteristics and are critical to addressing potential conflicts and contradictions and achieving coherence.	In dyad leadership, two persons, a manager (DCL) and a physician/medically trained individual (PL), share the responsibilities. In some instances, the administrative leader has a nursing or technical background. To achieve organisational goals, the two leaders in the dyad leadership model usually have different primary areas of responsibility.
St. Fleur, R.; McKeever, J.	The Role of the Nurse‐Physician Leadership Dyad in Implementing the Baby‐Friendly Hospital Initiative	2014	Nursing, Medicine	United States (New Jersey)	Descriptive case study	This study examines the implementation of the Baby‐Friendly Hospital Initiative (BFHI) through the collaborative efforts of nurse physician leadership dyads. The authors describe the processes, challenges and outcomes associated with this leadership model in this specific healthcare setting. By detailing the experiences and strategies employed, the article provides insights into how the nurse physician dyad model can facilitate significant cultural and practice changes within maternity care.	The authors use the definition of a dyad of Zismer and Brueggemann (2010). ‘A dyad are two persons involved in an ongoing relationship or intervention; the relationship or intervention itself’.
Steinert, T.; Goebel, R.; Rieger, W.	A nurse‐physician co‐leadership model in psychiatric hospitals: results of a survey among leading staff members in three sites.	2006	Nursing, medicine	Germany	An evaluation of a physician–nurse leadership model based on anonymous interviews with a questionnaire containing 45 items, conducted with all of the 165 leading staff members regarding their satisfaction with this new leadership model.	Overall, the leading staff members were satisfied with the shared leadership model. Nonmedical staff members were significantly more in favour of several aspects of shared leadership than physicians. The results provide some evidence that the shift from traditional leadership models to the physician–nurse shared leadership model may have advantages in the management of psychiatric hospitals.	Shared leadership model of medico‐therapeutic staff and nursing staff was not explicitly defined.
Terwilliger, I. A.; Johnson, J. K.; Manojlovich, M.; Astik, G. J.; Kim, J. S.; Williams, M. V.; O′Leary, K. J.	Contextual Factors Influencing the Implementation of a Multifaceted Intervention to Improve Teamwork and Quality for Hospitalized Patients: A Multisite Qualitative Comparative Case Study	2024	Nursing, medicine	United States	A multisite qualitative comparative case study to identify factors associated with successful implementation of a set of complementary interventions to redesign clinical microsystems. This Advanced and Integrated MicroSystems (AIMS) interventions include unit‐based physician teams, unit nurse–physician co‐leadership, enhanced interprofessional rounds (IPR), unit‐level performance reports, and patient engagement activities. 4 US hospitals participated in the RESET study.	Four contextual factors were associated with implementation success: senior hospital leader involvement and organisational support; alignment of RESET with organisational, hospital and professional group priorities; site leaders’ engagement in RESET and relationship with one another; and perceptions of need and intervention benefits among professionals.	Unit nurse–physician co‐leadership: collaborative model in which a nurse leader and physician leader are jointly responsible for quality performance on their unit
Thude, B. R.; Thomsen, S. E.; Stenager, E.; Hollnagel, E.	Dual leadership in a hospital practice	2017	Nursing, medicine	Denmark	Qualitative study: semistructured interviews to clarify how dual leadership works in a hospital context. Six leaders from 3 dual leader teams from the same ward. Analysis: all interviews transcribed and coded with coding focus on 9 principles used in the Roman Republic, added 1 extra principle bij looking at generic themes	Differences in the three teams show that just having dual leadership is insufficient to make it work. Power balance, personal relationships and decision process are important factors in dual leadership.	Dual leadership is defined as a setting where two leaders are mandated without any power difference or specified task division to have executive roles or duties and are held jointly accountable for the company’s or unit’s results. Refer to the definition of Denis et al (2012): dual leadership is a subset of pooled leadership, defined as two or more leaders working as co‐leaders. Dual leadership is often found in knowledge‐based organisations where a team of leaders mutually lead others.
Trask, M.; Webb, M.; Dickson, G.; De Gagne, J. C.	Learning together: A quality improvement project on tandem training for dyad leadership partners in healthcare	2025	Healthcare	Canada (Ontario)	Pre‐post design to evaluate the impact of a self‐directed educational intervention using the LEADS framework. Satisfaction data were collected through anonymous interviews and self‐assessment date were collected pre‐ and post‐intervention using the LEADS self‐assessment tool.	An 80% satisfaction rate was achieved for the relevance of the presentation, content clarity and overall rating, from 5 respondents of the 14 participants. Self‐assessment scores indicated significant improvements in self‐awareness and managing‐self scores with 20%, developing‐self with 33,3% and demonstrating‐character scores with 9 %. 50% completed the self‐assessment tool.	Dyad leadership occurs when a clinical leader, often a physician, is paired with an administrative leader who is often a nurse or allied health professional. Together they have joint leadership responsibilities for a team or a program within an organisation.
Warren, J. B.; Wiggins, N.	Shared Decision Making in Neonatal Quality Improvement	2016	Nursing, medicine	United States (Oregon)	Descriptive case study	The authors describe an ongoing quality improvement project in the Doernbecher NICU, highlighting factors contributing to its early success. These include the NICU’s history of quality improvement, the ‘dyad leadership’ structure and a growing understanding of team intelligence. Together, these elements have helped create a team that practices shared decision‐making and works toward a common goal.	The authors use the definition of Sanford and Moore (2015) and state that a dyad leadership management structure is defined by a unique collaboration between nursing and physician leaders who have shared goals and vision.

#### 4.1.3. Grey Literature

To ensure a broader and more comprehensive understanding of the concept, the search was expanded to include grey literature. This enabled the identification of additional sources of information not available in the consulted online databases. These sources included organisational websites, professional reports, educational materials, practitioner‐orientated publications (e.g., professional periodicals), and multimedia sources such as a podcast.

A report published on the organisational website of the ‘American Organization for Nursing Leadership’ (AONL) describes a model of clinical partnering based on the concept of dual leadership, in which nurse and physician leaders collaborate as equal partners. The model emphasises the importance of relational aspects such as trust, communication and mutual respect and highlights synergy as a key strategy for achieving shared goals and improving patient outcomes [47]. Another source from the grey literature is the article ‘Duaal leiderschap in beeld’ (Dual Leadership in Perspective) by Anne‐Marie Poorthuis, published in ‘Eigentijdse Verbindingen’ [48]. Poorthuis describes dual leadership as a model in which a nurse and a medical leader jointly manage a clinical unit. The article emphasises the integration of different professional perspectives into a shared leadership framework, rather than a mere division of tasks. Effective dual leadership is presented as highly dependent on relational factors such as trust, communication and mutual adaptation. While the model is considered promising for creating integrated care environments, the author also highlights its challenges, including the risk of parallel rather than truly collaborative leadership and the importance of interpersonal ‘chemistry’ between partners [48].

A podcast titled ‘Samen sterker: de essentie van duaal leiderschap in de zorg’ (Stronger Together: The Essence of Dual Leadership in Healthcare) further elaborates on the concept of dual leadership in healthcare. Van ’t Geloof [49], a business economist and lecturer with extensive experience in studying collaborations between physicians and managers since 2012, describes dual leadership as a model in which a managerial leader and a medical leader jointly influence two interconnected domains of value creation: An organisational or managerial domain and a clinical or medical domain. In this model, both domains are considered equally central, with the patient as the overarching focal point. The podcast suggests that dual leadership is applied in various international healthcare contexts and describes it as being operationalised through different models and practices that aim to integrate managerial and clinical perspectives within a shared leadership framework. The podcast is classified as grey literature due to its nonpeer‐reviewed and practice‐orientated nature.

To address both the semantic variation and the conceptual ambiguity present in the literature, a new definition was developed by synthesising the identified defining attributes, the terminology and variations found in the literature, and contextual insights drawn from healthcare‐related sources. This integrative approach ensures that the definition reflects both the conceptual essence of NPDL and its practical relevance within clinical settings.

Based on this synthesis, the following definition is proposed:

*Nurse–physician dual leadership is defined as a collaborative leadership model in which two formal leaders, a nurse leader and a physician leader, work together as co-leaders, with different skill sets and without a power difference, and they are jointly accountable and responsible for the organisational or unit results*.

### 4.2. Defining Attributes

The central aim of concept analysis is to identify the defining characteristics of a concept. Recurring characteristics identified in the literature are grouped to highlight the features that distinguish the concept from related or similar phenomena. Walker and Avant [38] define attributes as characteristics that appear repeatedly in a concept and help researchers differentiate the occurrence of a specific phenomenon from a similar one. Based on the literature, NPDL is characterised by four key attributes: a formalised dual leadership structure, joint responsibility, joint decision‐making and role complementarity. Together, these attributes capture the core features that distinguish NPDL as a collaborative leadership model in acute hospital settings.

#### 4.2.1. Formalised Dual Leadership Structure

A formalised dual leadership structure constitutes the foundational attribute of NPDL. Without formal organisational embedding and recognition of both leadership roles, the concept of NPDL cannot exist. In an NPDL team, the nurse and the physician hold formal management positions within the organisation, and together they lead and manage a programme, department, or division of care [50]. The leaders of the team are recognised leaders within the organisation [10, 13]. They influence each other and have a collective impact on their followers [13].

Therefore, the dual leadership model should be embedded in the organisation’s organogram, with clearly defined communication lines within the formal communication structure [50, 51]. Ideally, both leadership partners are positioned at equivalent hierarchical levels within the organogram, reinforcing their shared authority and collaborative function [12]. A joint reporting structure aligns with the overall shared accountability and responsibility of the partners and can help resolve disagreements while presenting a unified front [14]. Ideally, a higher‐level dual leadership team provides supervision and strategic guidance to the nurse and physician leadership pair [14]. This team usually comprises senior representatives from nursing and medical management, reflecting the same interprofessional collaboration across multiple higher levels in the organisation. Nevertheless, the extent to which NPDL is implemented across multiple hierarchical levels varies depending on the specific organisational structure and management tradition. Some authors suggest that, within a formalised dual leadership structure, it is preferable to place the dual leaders in adjacent offices, if possible. This proximity enhances informal collaboration, allowing the partners to become sounding boards for each other quickly, and supports regular meetings [1, 4, 34].

#### 4.2.2. Joint Responsibility

Joint responsibility represents the second defining attribute of NPDL. Without shared responsibility for significant outcomes, NPDL cannot be distinguished from parallel or discipline‐specific leadership arrangements. In NPDL, the two leaders share executive leadership roles and functions and are each responsible and held accountable for clearly defined domains within the organisation, with a clear distinction between shared and individual responsibilities [9, 12, 14, 19, 52]. They usually have different primary areas of responsibility to support the achievement of organisational goals effectively [14]. Sanford and Moore [12] describe this as having distinct job descriptions and complementary roles that support one another in practice. NPDL is an ongoing, dynamic, and context‐dependent process that develops as the organisational culture evolves [9]. The leaders actively seek and design opportunities for collaboration, resulting in shared responsibility and accountability for the outcomes of significant issues [1].

Thude et al. [4] suggest that task division within dual leadership structures may occur organically or be shaped by the specific context. Task division here refers to the allocation of specific tasks, along with their associated responsibilities, between dual leaders, for example, one focusing on nursing management and the other on clinical decisions. Given the collaborative nature of dual leadership, some tasks and responsibilities are also shared. Other authors also emphasise that clearly delineated roles are essential for effective collaboration within dual (and triadic) leadership teams [20, 53].

#### 4.2.3. Joint Decision‐Making

A key aspect of this collaborative relationship is how decisions are made. The leadership team is responsible for joint decision‐making [14], and they must agree on the decision‐making process [4]. A clear and balanced strategy of shared decision‐making can play a crucial role in preventing the emergence of conflicts [2, 12]. For dual leadership to be effective, both partners must be willing to relinquish a degree of their individual professional autonomy to enable shared decision‐making and joint accountability [53], where the patient remains the primary focus. Joint decision‐making therefore constitutes the third core attribute of NPDL.

Identifying discipline‐specific domains does not mean that decisions take place in isolation from other colleagues [9]. According to Ponte [9], understanding the lines of authority and accountability is a crucial first step in establishing an effective interdisciplinary governance model. Consequently, dual leaders need to recognise the scope of their decision‐making and determine which decisions fall under shared versus distinct accountability. In practice, this means that leaders may independently make decisions within their discipline‐specific domains while informing and consulting one another when decisions affect shared responsibilities or the broader interprofessional team [4, 9].

#### 4.2.4. Role Complementarity

The fourth defining characteristic of NPDL is the complementary nature of the leadership roles. The leadership partners bring diverse and specific perspectives, with the goal of leveraging each other’s strengths to improve organisational performance and patient outcomes [8, 9, 34]. Effective collaboration occurs when the partners are explicit about their expectations and goals [53, 54] and work together as a unit [10].

An organisation can benefit from the complementary skill sets brought by pairing two leaders with different backgrounds [2]. According to Collins et al. [2], leadership selection is a critical factor in the success of dual leadership. Clausen et al. [50] describe this as ‘intentional partnering’, where leaders are purposefully paired to leverage their complementary skills and strengths to enhance leadership effectiveness.

In the context of NPDL, the complementarity of individual skills between the partners—and the compatibility of these skills and their leadership styles—play a crucial role in the success of the partnership [20, 23, 34]. Together, the nurse and the physician leader can synergistically develop a full spectrum of leadership skills [37, 53] by complementing each other’s strengths and covering each other’s weaknesses [13, 53]. A suitable leader pairing should share enough qualities to align on goals and operations yet differ sufficiently so they can divide responsibilities and introduce diverse perspectives into leadership processes [13]. Once a partnership is established, the nurse and physician can draw on each other’s strengths to work effectively together to address patients’ interests [50].

As a result of leaders’ complementary skills and leadership styles, their collaboration is often more effective in implementing initiatives and driving organisational goals. Therefore, it is critical to dedicate time early in the partnership to develop a shared understanding of each other’s strengths and weaknesses [1]. Leadership development interventions, focusing on both the individual and the partnership, can play a crucial role in supporting the development of strong and effective dual leadership [1, 20, 53].

According to Iannazzo et al. [37], postgraduate nursing education provides nurse leaders with the competencies necessary to facilitate multidisciplinary team functioning and drive clinical and quality improvement initiatives in partnership with physicians. In contrast, medical education has traditionally focused on biomedical expertise and individual performance, which may contribute to limited formal training for physicians in teamwork and interdisciplinary collaboration. Within NPDL, these differing educational backgrounds contribute to role complementarity: The nurse leader brings expertise in leading multidisciplinary teams in complex clinical settings, while the physician leader holds the unique ability to engage medical peers, together forming a powerful and complementary leadership force. This underscores why a nurse leader is the ideal partner within a dual leadership model, uniquely positioned to collaborate with the physician leader.

### 4.3. Model Case

A model case of a successful dual leadership pair embodies all the defining attributes of the concept [38]. The following hypothetical case illustrates this. Mr. Hayes, head nurse of the emergency department, and Dr. Carter, emergency doctor and medical director of this department, jointly lead the unit and the multidisciplinary team within a formalised leadership structure. Their leadership is characterised by joint responsibility for the overall functioning of the emergency department. While Mr. Hayes oversees the nursing staff, operational processes and patient flow, Dr. Carter is responsible for medical care, protocols and patient treatment. Together, they monitor the overall effectiveness of the unit, regularly reporting results to their supervisors. Joint decision‐making is central to their collaboration. Mr. Hayes and Dr. Carter always consult each other first before communicating the decisions to the team and agree on the extent to which the multidisciplinary team, including nursing and medical staff, can participate in decision‐making. Their skills complement one another, with Mr. Hayes excelling in managing personnel and operational processes and Dr. Carter bringing extensive medical expertise. The support they provide to each other strengthens their leadership roles. This model case exemplifies the defining characteristics of a successful dual leadership structure, in which joint responsibility, joint decision‐making, and role complementarity are crucial for delivering high‐quality care and achieving team success.

### 4.4. Borderline Case

A borderline case of NPDL encompasses several key elements of the concept but does not fully meet all of its defining criteria [38]. For instance, consider the hypothetical case of Mr. Hayes and Dr. Carter, the formal dual leaders in the emergency department of a general hospital. Although they collaborate based on the principles of dual leadership and draw on each other’s expertise, their dual leadership structure is not formally established within the organisation, nor is it reflected in the organisational chart. Joint decision‐making is inconsistent, as they make use of each other’s expertise, but they only discuss the difficult cases with each other. As a result, within their collaboration, they cannot take full joint responsibility for the outcomes of the emergency department. While this borderline case includes several defining elements of NPDL, the lack of formal structural integration and consistent joint decision‐making prevents it from fully exemplifying the concept.

### 4.5. Contrary Case

Once again, the hypothetical case of Mr. Hayes and Dr. Carter is used to illustrate a contrary case. A contrary case of NPDL lacks all the essential characteristics of the concept [38]. Although Mr. Hayes and Dr. Carter have been introduced to each other as dual leaders, no formalised dual leadership structure exists within the organisation. They rarely consult with each other and spend almost no time together, failing to utilise each other’s expertise, resulting in an absence of joint decision‐making. Instead, the nurse leader, Mr. Hayes, tends to make decisions quickly without consulting Dr. Carter. As a consequence, there is no shared responsibility for the overall results of the emergency department. Furthermore, their professional relationship is characterised by a lack of trust and mutual support. This prevents them from utilising any form of role complementarity. Rather than reinforcing each other’s leadership, they act in isolation and impede each other rather than strengthen one another. This contrary case demonstrates the complete absence of the defining attributes of NPDL.

### 4.6. Antecedents

Antecedents of NPDL must be in place before the concept emerges [38]. A review of the literature identified five antecedents of NPDL, which are divided into organisational and relational factors. Organisational factors include a supportive organisational culture and leadership role space. Relational factors include the ability to trust, the will to collaborate and the ambition to share. The context of NPDL is typically an organisational setting characterised by interprofessional care teams, consisting at a minimum of nurses and physicians, as the model is embedded in collaborative care environments where nurses and physicians work within shared team structures. The interprofessional care teams could potentially include other healthcare professionals [54].

#### 4.6.1. Supportive Organisational Structure and Culture

A supportive organisational structure and culture are essential for the development and sustainability of NPDL [1, 2, 9, 12, 34, 50, 54, 55]. The organisation must clearly articulate the rationale behind this approach, not only to the leadership partners but to all internal stakeholders [8]. The leadership relationship should be acknowledged, supported and expected by the other leaders within the organisation [1]. Securing buy‐in from senior clinical leaders, who can serve as change champions and support the model, is also crucial [53].

The organisation must also be willing to invest in extensive training programmes that provide both leaders with the necessary skills and competencies to function effectively within a dual leadership structure [20]. The CNO‐CMO partnership at the system level can act as a model for similar collaborations throughout the organisation [1].

Furthermore, it is the responsibility of the organisation to provide a robust data system that enables dual leadership teams to make informed decisions, report internally to supervisors and team members, and report externally to relevant regulatory and oversight bodies. Healthcare both uses and generates data for nearly every aspect of the care delivery process. Data are crucial to evaluate the added value of decisions or the impact of improvement initiatives. Healthcare organisations must also report data to assess performance, both internally and externally. Typical metrics include service volume, clinical quality outcomes, financial performance, patient experience scores and staff‐related outcomes [12]. In doing so, they can jointly take ownership of their responsibilities and be held accountable for the outcomes of their decisions and actions.

#### 4.6.2. Leadership Role Space

Leadership role space refers to the formal organisational structural and contextual conditions that create and protect the possibility for nurse and physician leaders to enact joint leadership roles, ensuring that leadership authority is distributed and not concentrated in a single individual [12, 22]. It encompasses the structural definition and institutional positioning of the dual leadership arrangement, rather than cultural or relational aspects. Within NPDL, leadership role space requires clearly delineated yet complementary leadership roles, supported by an explicit and shared mandate [14]. Both leaders must be formally authorised to assume leadership responsibilities and exercise decision‐making power within their joint responsibility domain [9, 12, 19, 52]. This role clarity prevents ambiguity regarding authority and accountability and distinguishes dual leadership from advisory or consultative arrangements. Within a dual leadership structure, it is essential that the leadership relationship is formally recognised and supported by other leaders in the organisation and that it is embedded within the organisational leadership structure [12, 14]. Additionally, the presence of a high‐functioning professional in a support role is vital to ensuring the effectiveness of nurse–physician leadership teams [9].

#### 4.6.3. Ability to Trust, the Will to Collaborate and the Ambition to Share

Because dual leaders are required to cultivate a strong mutual professional and personal relationship [13, 50, 54], they must have the ability to trust. This relationship is based on trust, respect and authenticity [2, 10, 50]. These factors are fundamental to establishing psychological safety, which is critical for creating a fertile ground for sharing knowledge [19] and feeling confident in understanding and anticipating each other’s behaviours and intentions [50]. This mutual trust enables the direct and open exploration of issues [35] and is essential; neither member of the partnership should dishonour a shared agreement retrospectively [1]. Communication and interpersonal relationships appear to form a mutually reinforcing loop. Honest and timely communication both require and build trust, credibility and respect [50].

In addition, the leadership partners must have the will to collaborate, as without this disposition, no foundation for NPDL can be established. Nurse and physician leaders need to learn and develop capacities and competencies to work in partnership and co‐lead across their clinical and management roles. The effectiveness of NPDL is likely based on each partner’s contribution to the partnership [19] and their commitment to it [2, 9, 35, 42]. Ponte [9] also refers to the importance of a shared understanding of interdisciplinary leadership and how the joint leadership will be most effective. Commitment in such partnerships goes beyond a general willingness to collaborate and requires an active investment of time, effort and energy to build and sustain the partnership. In this context, Tjosvold and MacPherson [56] refer to the concept of ‘constructive controversy’, which describes an open‐minded discussion within a strong cooperative context, where various perspectives allow for disagreement and exploration in a respectful manner. Therefore, building productive partnerships based on shared values between leaders is crucial for success [19, 50, 51].

The ambition to share power, knowledge and successes is another crucial antecedent that must be present prior to engaging in dual leadership. An effective dual leadership team must maintain a healthy power balance [4], in which both leaders are prepared to compromise and express humility [1, 12, 50]. Mutual respect as equal partners is essential, ensuring that both contribute equally to decision‐making and the achievement of organisational goals [1]. Cultural differences between physicians and formal leaders in an organisation that result in interdisciplinary relationship problems and conflicts have been documented in the literature for over a century. Power struggles between the two groups have long been recognised [12]. Power sharing—which includes shared leadership at the CEO, ward and clinic levels—throughout the entire organisation—can be a lever for success [4]. Dual leaders must also be willing to share knowledge, as this is essential for integrating their complementary expertise and enabling effective joint decision‐making within NPDL. Leaders who learn together, grow together and expand their understanding of themselves and each other [1, 15]. The dual leadership partners must recognise and value each other’s position of influence [50], accept the duality and be humble enough to share successes [4]. Their joint influence will only be effective if the two leaders truly act as partners with aligned visions and goals [12].

### 4.7. Consequences

The implementation of NPDL has been described in the literature as being associated with four consequences. These consequences include stronger leadership, enhanced interprofessional collaboration, improved quality of decision‐making and forming a united leadership front. The literature also indicates that inadequate preparation, implementation, or sustainability of NPDL may be associated with potentially negative consequences.

#### 4.7.1. Stronger Leadership

Within NPDL, the two leaders can learn from each other, bridge gaps and enhance the organisation’s overall performance that neither could achieve on their own [2]. As such, NPDL may contribute to stronger leadership. This is further supported by opportunities for continuous mutual learning [34]. Moreover, according to Biga [20], leadership continuity and enhanced professional development are key benefits of the dual leadership model. In addition, dual leadership can help reduce the cognitive and emotional workload and the responsibilities of individual leaders by distributing key leadership functions [13]. Consequently, it supports leaders’ well‐being, as leading is cognitively demanding and involves numerous stressors. Finally, to continuously develop both themselves and the relationship between the two leaders, it is crucial that the leadership partners are allocated protected time for reflection and joint growth [1, 17, 19, 49].

#### 4.7.2. Enhanced Interprofessional Collaboration

Dual leadership offers the opportunity for enhanced collaboration, improved teamwork [15], and continuous learning among healthcare professionals [20]. The leaders can serve as role models for the behaviours and changes they wish to achieve [9, 10]. At all levels, leadership partners can maintain stability and success by fostering interprofessional cooperation and reinforcing their collaborative relationships. Mutual respect, trust and teamwork are key factors in achieving this [51].

Dual leadership can help optimise resource utilisation and strategic planning [20]. Its shared vision encourages systems thinking, ensuring that organisational resources are optimally aligned and utilised to enhance efficiency, effectiveness and ultimately improve outcomes [51]. Other benefits of enhanced interprofessional collaboration include increased engagement, improved accountability and performance, reduced leader burnout [12] and enhanced retention in leadership roles [15]. An effective implementation of NPDL in today’s healthcare organisation can systematically create an aligned organisation culture and help bridge the gap between historically siloed practices [12]. Dual leadership provides an opportunity for more streamlined communication across interdisciplinary teams, including between nurses and physicians [12, 14, 20, 50, 54]. Enhanced communication and collaboration can help reduce existing siloes [50].

#### 4.7.3. Improved Quality of Decision‐Making

The joint expertise of the two leaders enables more informed decision‐making [8–10, 13] and leads to quicker, more effective choices [20]. Two leaders have a greater span of influence than one. In challenging and rapidly changing times, this greater span of influence, resulting from a leadership team with leaders from two different professions and professional cultures, is one of the most significant benefits [12]. As a result, dual leadership facilitates a broader support base within the organisation and contributes to faster and greater acceptance of decisions. This collaboration enhances joint problem‐solving and the achievement of shared goals [50, 54], while helping to increase patient outcomes and organisational efficiency [15].

Quality improvement can be a potential benefit of implementing dual leadership [20]. Joint decision‐making is an inherent attribute of NPDL and enhances the quality of decision‐making if it is effectively enacted. This leads automatically to the improvement of quality of care. NPDL can also catalyse the initiating and sustainability of improvements by enhancing morale, engagement and investment among all care providers, ultimately driving quality and safety initiatives [55]. An effectively collaborating nurse–physician leadership duo can drive practice‐based research that supports healthcare quality improvement, representing a significant and positive outcome of this leadership model [14].

#### 4.7.4. United Leadership Front

Within the dual leadership structure, ongoing professional development is necessary. Interpersonal skills have been identified as important competences and require a ‘growth mindset’ [19]. To provide effective leadership and create an interdependent and effective duo, it is essential for leaders to be able to manage themselves. The mental model needs to evolve into a more expansive and inclusive mindset [19], where joint accountability demands a united front. The principle of ‘never speaking ill of the partner’ exemplifies the mutual respect and trust that exist between dual leaders [1, 52]. Despite the presence of two leaders, they are expected to present a unified voice and act in concert within the partnership, consistently demonstrating this cohesion to the broader organisation [1, 2, 10].

As part of this collaborative mindset, within the NPDL structure, the leadership partners need to develop an identity as ‘a leadership unit’ [13]. This shared identity is crucial, as their mindset must be permanently orientated towards collaboration, mutual respect and shared responsibility. These principles should be a constant and integral aspect of their leadership approach, to ensure that they consistently prioritise the collective short‐ and long‐term goals of the organisation, the team and patient care. They must lead in a manner that continually honours the mission and the vision of the organisation [12].

Figure 1 illustrates a model summarising all the key attributes, antecedents and consequences of NPDL.

### 4.8. Empirical Referents

Identifying the empirical referents for its defining attributes is the final step in analysing. Empirical referents are observable classes or categories of phenomena whose presence indicates the existence or occurrence of the concept in practice. However, they relate directly to the defining attributes of the concept. Empirical referents are not measurement tools for assessing the concept itself. They function as observable indicators of how the concept appears in real‐world contexts and thus serve as a bridge between its abstract and theoretical formulation and its practical measurability [38].

Importantly, NPDL can be conceptually distinguished from other forms of shared or collaborative leadership. While many shared leadership models generally emphasise the distribution of leadership influence across team members [22], NPDL is characterised by a formalised dual leadership structure, joint responsibility, joint decision‐making and role complementarity. These four defining attributes must be translated into observable indicators that reflect their presence in clinical settings.

A formalised dual leadership structure provides the organisational foundation necessary to enable the enactment of joint responsibility and joint decision‐making. This structure can be observed through organisational configurations that clearly define governance arrangements and formalise roles, authority and decision‐making responsibilities, thereby explicitly embedding shared leadership between a nurse leader and a physician leader. This structural dimension becomes visible through the formal assignment of NPDL roles in organisational charts [50, 51], with both leaders positioned at the same hierarchical level within a clinical service, division, department, or the organisation as a whole [12]. In addition, the formalised dual leadership structure is reflected in the composition of organisational committees or governing bodies related to key domains for which joint responsibility is expected, such as quality and safety committees, clinical governance boards, or strategic steering groups. The inclusion of both nurse and physician leaders in these structures further evidences their shared authority and accountability in practice.

Joint responsibility can be identified through shared accountability mechanisms for strategic, organisational and clinical outcomes. Observable referents include jointly developed policy and strategic plans, linked performance objectives, and project or innovation plans that reflect shared responsibility for service‐level outcomes. In addition, jointly owned clinical protocols and care pathways may further evidence the formalisation of shared responsibility in practice. Furthermore, joint responsibility may be formalised in position descriptions or mandate documents that explicitly distinguish between individual and shared domains of accountability, whereas responsibilities are clearly identified and understood [12]. At the process level, joint responsibility becomes visible through co‐accountability in quality improvement plans, evaluation processes and reporting meetings, as well as through the use of shared performance dashboards and other integrated reporting instruments that reflect shared responsibility for outcomes and continuous quality improvement.

The joint decision‐making attribute is reflected in collaborative processes in which both leaders actively participate in and influence key decisions. Empirical referents include co‐chaired meetings [14], documented shared approval of strategic and operational decisions, and structured consultation processes between both leaders. In addition, formally established decision‐making procedures that require mutual input prior to implementation further indicate the presence of joint decision‐making. This process may be supported by a formalised framework that explicitly defines roles, responsibilities and decision‐making modalities, including clearly articulated escalation mechanisms for situations in which consensus cannot be reached. Minutes of operational or management meetings in which joint decision‐making is documented provide additional empirical evidence. The presence of joint decision‐making can further be recognised through the alignment between decision‐making practices and formally defined individual and shared domains of accountability [12, 14].

Role complementarity can be observed through consistent patterns of differentiated yet aligned leadership contributions across clinical and organisational domains. Rather than being expressed through fixed task allocation, it becomes visible in the way both leaders systematically draw on distinct professional expertise while maintaining alignment in decision‐making, communication and strategic direction. This complementarity is further reflected in the dynamic enactment of leadership roles in practice, including the way responsibilities are distributed across specific projects and strategic objectives, the leadership of meetings or agenda items depending on contextual expertise, and the extent to which each leader assumes leadership functions in line with situational demands. In addition, role complementarity can be identified in recurring patterns of implicit role‐taking, whereby nurse and physician leaders alternately or jointly assume responsibility for initiating, coordinating, or synthesising actions while ensuring a coherent and unified leadership direction. These recurring patterns of differentiated yet aligned leadership contributions provide observable indicators through which role complementarity can be empirically recognised in practice [22].

While no validated instruments currently exist to measure NPDL as a distinct construct, these observable manifestations translate the defining attributes of NPDL into empirically identifiable indicators, providing a practical basis for recognising and studying NPDL in clinical settings.

## 5. Discussion

Terminologies and definitions used in the literature vary significantly to describe a collaborative leadership structure in which two formal leaders work together as co‐leaders. These leaders bring different skill sets, operate without a hierarchical power structure, and share joint accountability and responsibility for the outcomes of the organisation or unit. Among the various terms used—such as dual leadership, co‐leadership, dyad leadership and other related concepts—the term *dual leadership* is chosen to refer to the concept as envisioned and described in this concept analysis.

Within the healthcare literature, dual leadership is described in two main constellations: one constellation as a partnership between a nurse leader and a physician leader [1, 9, 37, 51, 54, 57]; another constellation as a partnership between a physician leader and an administrative leader [8, 20, 34], who may, in some cases, have a nursing background [19]. Nevertheless, nurses and physicians are two key stakeholders in healthcare, each bringing distinct expertise and professional cultures. They are uniquely positioned to contribute their knowledge and expertise to the important decisions that must be made daily within healthcare organisations, particularly because they represent two essential professions that deliver patient care 24/7. Although the nursing shortage is a harsh reality and a significant threat to healthcare [58], this profession must be strongly represented at all levels of healthcare organisations and healthcare policy [59, 60]. This highlights the critical importance of collaboration between these professions across all organisational levels, ensuring that the nursing perspective is consistently represented in decision‐making processes. Maintaining this representation is essential to avoid the marginalisation of nursing voices in the future.

What is clear is that in order to successfully implement NPDL, thorough preparation is required, both at the organisational level and on an individual and partnership level. Strong organisational support, along with an appropriate organisational structure and culture, is essential. Aligning vision and objectives, ensuring a good fit between the two leaders, and building a lasting personal and professional relationship are indispensable success factors of dual leadership. All of this must be built on fundamental values such as respect, honesty and authenticity. However, the question arises whether NPDL should always be part of a set of interventions that collectively contribute to a sustainable system. Within the explored literature, authors referred often to this model as one of the strategies to reform the current microsystems into more efficient, collaborative and patient‐centred structures that are future‐proof and capable of adapting to the evolving needs of healthcare [17, 47, 56, 57].

Within the scientific literature, a consensus can be found regarding the attributes and antecedents of dual leadership or within the synonyms used for this concept. However, there is a lack of consensus about the appropriate level of clarity in task and role distribution within dual leadership. Some authors emphasise the importance of clearly delineated responsibilities [9], while others advocate for a more flexible and shared approach to leadership roles. In addition, few authors remain vague on this matter, describing dual leaders as having the same [34] or even identical job descriptions and reporting structures, without specifying how responsibilities are divided in practice. This variety of perspectives reflects the complexity and dynamic nature of dual leadership, highlighting the need for adaptation to the specific context and the needs of the team.

The organisation‐wide implementation of NPDL entails more than an alternative leadership model. It fundamentally transforms the traditional unitary hierarchy, with its clear hierarchical lines, information flows, and implicit power structures, into a system of shared leadership that redistributes roles and responsibilities. This model offers a great opportunity for authority to be jointly exercised while formal hierarchy remains. Such a paradigm shift affects the formal organisational structure, requiring revisions to existing organograms and decision‐making bodies, while simultaneously challenging the organisational culture to evolve towards greater collaboration, shared decision‐making, and mutual understanding across professional groups. For leaders, NPDL can broaden perspectives and enrich decision‐making, yet it may also constrain traditional autonomy, thereby necessitating new competencies, redesigned leadership development programmes, and even curricular adjustments in health professional education. At the team level, NPDL reshapes dynamics through altered coordination mechanisms, enhanced interprofessional collaboration, and role‐modelling by leaders, all of which influence team functioning and responsibility‐taking and team climate. The critical question for future research is how hospitals might redesign their structures, cultures, leadership practices, and team arrangements to redistribute power and responsibility while maintaining clarity, accountability, and effective decision‐making within an NPDL‐based shared leadership system.

A successful implementation of NPDL can enhance decision‐making quality [8, 9], improve collaboration [15, 50] and continuous learning among healthcare professionals [2, 20], optimise resource utilisation and strategic planning [20], and contribute to improved quality and safety of care [15, 20]. It can also positively affect the work environment for leaders and teams [13, 53], reduce leadership burnout [12], support retention in leadership roles [15], and foster personal and professional development [20]. Conversely, if NPDL is poorly managed, it can increase the potential for conflict, create gaps between nurses and physicians, negatively impact the work environment and the well‐being of leaders and team members, and lead to delays or reduced focus and clarity [4]. Therefore, careful consideration of the organisational context, commitment and readiness of the dual leaders and their foundational competencies is essential to realise the benefits and mitigate the risks associated with NPDL.

International perspectives are important when considering NPDL implementation, as cultural and contextual factors may influence feasibility and effectiveness. In many European countries, implementation may be facilitated by established structures for interprofessional collaboration [61], advanced leadership education, professional autonomy and supportive policies. In contrast, implementation in LMICs may encounter additional challenges, including more hierarchical organisational cultures, limited resources [62], and variability in professional training pathways [63]. These contextual differences suggest that strategies for NPDL adoption need to be adapted to local organisational, cultural and educational realities.

This concept analysis has several methodological limitations. First, the search was limited to three databases (PubMed, Embase and ScienceDirect), which may have resulted in selection bias, even though they cover the majority of clinical and organisational research. Second, the search was restricted to publications in English, which may have excluded relevant studies published in other languages. Third, although broader leadership terminology was initially explored, it was excluded to maintain conceptual precision; nonetheless, it is possible that some relevant insights were missed. Fourth, empirical evidence on formal dual leadership between nurse and physician leaders remains limited, constraining the generalisability of findings.

Additionally, the use of grey literature, while valuable for capturing emerging perspectives, entails variability in methodological quality. Nonetheless, the included studies provide a coherent overview of the core evidence and highlight recent developments and interest in formal dual leadership models in hospital settings. Together, these limitations may influence the comprehensiveness of the concept map; however, the point of conceptual saturation, combined with repeated iterative searching, strengthens confidence in the robustness of the derived attributes, antecedents and consequences.

Finally, while conceptual saturation was reached within the included literature, further refinement and validation of the concept of NPDL would benefit from qualitative research, conducted from a multilevel perspective, which could provide richer contextual insights and explore the perspectives of practitioners directly involved in these leadership roles.

## 6. Conclusion

The NPDL approach holds promise as a potential solution to the complex challenges facing healthcare. For successful implementation and evaluation, a clear conceptualisation is essential. The resulting model offers a structured understanding of NPDL and may support hospital management, policymakers, educators, facilitators, and researchers in advancing collaborative leadership practices.

NPDL is defined as a collaborative leadership model in which two formal leaders, a nurse leader and a physician leader, work together as co‐leaders, with different skill sets and without a power difference, and they are jointly accountable and responsible for the organisational or unit results. The definition and the clarified concept provide hospital management with a clearer basis for designing and supporting effective nurse–physician leadership pairs. It also offers policymakers guidance for creating organisational conditions that enable and sustain dual leadership structures in clinical settings. Additionally, it enables educators and facilitators to align training and professional development with the identified attributes. For researchers, the model offers a foundational framework to operationalise NPDL in future studies.

For nurse–physician dual leaders, the clarified definition and conceptualisation provide a shared and common frame of reference for the leadership model in which they operate. This conceptual clarity strengthens recognition of NPDL as an established and explicitly defined model and may foster a more comprehensive understanding of the conditions required for successful implementation, the defining characteristics of the model, and the potential benefits, risks, and challenges associated with its application.

Further qualitative research is recommended to refine and validate the concept of NPDL. Future studies should also focus on the development of a validated NPDL measurement instrument, the evaluation of its impact on clinical and organisational outcomes, and the exploration of training and educational models to support the acquisition and enhancement of dual leadership competencies among nurse and physician managers. Additionally, further research could explore the potential of NPDL in reducing departmental silos, fostering interprofessional collaboration and creating more integrated and supportive work environments.

## Author Contributions

Ines Vercalsteren: conceptualisation and design of the concept analysis, data collection, analysis, interpretation, writing–original draft, writing–review and editing, validation and accountability.

Filip Haegdorens: conceptualisation and design of the concept analysis, data collection, analysis, interpretation, writing–review and editing, validation and accountability.

Erik Franck: conceptualisation and design of the concept analysis, data collection, analysis, interpretation, writing–review and editing, validation and accountability.

## Funding

No funding was received for this manuscript.

## Conflicts of Interest

The authors declare no conflicts of interest.

## Data Availability

All data supporting the findings of this concept analysis are available in the published literature cited in the manuscript.
